# Practical Medicine

**Published:** 1880-04

**Authors:** 


*Collaborators:*

DR. L. W. CASE, DR. R. TILLEY.

### PRACTICAL MEDICINE.

ON ALCOHOLIC VASCULAR TENSION AND ITS EFFECTS.—(By John C. Thorowgood, M.D., F.R.C.P.)—*Medical Press and Circular*, 21st January, 1880.

Persistent excess in the use of alcohol by a healthy person maintains a condition of extra pressure and strain on the small vessels, which at last finds relief in hæmorrhage. If the patient be somewhat on in years, and have weak degenerative vessels, then a condition of tightness and over-pressure is most dangerous. How often do we have to see a robust-looking man suddenly struck down by cerebral hæmorrhage? Probably he has had a good dinner and drunk liberally, and when the doctor arrives and finds the poor man senseless on the sofa, every one says that he had been congratulated only the day before, and told how well he was looking. Supposing the vascular tension induced by a too free use of alcohol does not at once issue in a bleeding, it appears to maintain a constant strain on the heart and vessels, which seriously impairs the nutrition of the structure, so that the weak and wasted coats of the blood-vessels become very prone to laceration and rupture—a condition well expressed by the popular term, "rotten from drink." Thus cirrhosis of the liver and granular disease of the kidney may be set going. The variability in the degree of blood-pressure, according as the patient is or is not under alcoholic influence, produces a singular irritability of the nervous system. Sleepless nights with palpitating heart, morning retching and coughing, and utter want of appetite for breakfast, are among a few of these effects. Want of decision on momentous points, impatience and recklessness, tinglings, numbness, and shooting pains, are other signs of disturbed innervation. The sudden withdrawal of all stimulus from those persons who have been living in a perpetual state of excessive tension and strain is followed by more or less collapse and sense of exhaustion and sinking, driving a few weak ones back to the drink as being their only safeguard from doing something dreadful. The best advice to give during this very painful and trying crisis is to encourage the patient to outlive it, by assuring him that it is but temporary, and to take care that he is well supplied with the best of nourishment, and with good fresh coffee when he feels especially down and low, while at the same time he takes a bitter tonic medicine; and, under this regimen and a fair amount of resolution on his own part, there comes in time a renewal of life and a restoration of nerve that is most satisfactory to witness.

CASE OF PRECOCIOUS HYSTERIA.—M. Ad. Henrot. (*Union Méd. du Nord-Est.*, Dec., 1879, p. 363.)

The author reports the case of a young girl, aged 12 years, who suddenly, without any known reason, had convulsive attacks, with suffocation, cough, barking and esophageal spasms.

The attacks were very frequent, and had existed twelve days when the author saw the case for the first time. Her physiognomy was excellent, and nothing in her general condition would give rise to a suspicion of so profound a disturbance of the nervous system. The entire series of antispasmodic medicines had been employed without any result. M. Henrot thought it advisable to make use of a tolerably energetic means which had proved of value in analogous cases. At the first nervous attack which the young hysteric had in his presence, he flagellated her unmercifully with a napkin dipped in cold water. The attack ceased at once. Since that, the nervous troubles have completely disappeared.

